# Magnetically-driven 2D cells organization on superparamagnetic micromagnets fabricated by laser direct writing

**DOI:** 10.1038/s41598-020-73414-4

**Published:** 2020-10-02

**Authors:** I. A. Paun, C. C. Mustaciosu, M. Mihailescu, B. S. Calin, A. M. Sandu

**Affiliations:** 1 Center for Advanced Laser Technologies (CETAL), National Institute for Laser, Plasma and Radiation Physics, 077125 Magurele-Ilfov, Romania; 2 Faculty of Applied Sciences, University Politehnica of Bucharest, 060042 Bucharest, Romania; 3 Horia Hulubei National Institute for Physics and Nuclear Engineering IFIN-HH, 077125 Magurele-Ilfov, Romania; 4 Faculty of Applied Chemistry and Materials Science, University Politehnica of Bucharest, 060042 Bucharest, Romania; 5 CAMPUS Research Center, Doctoral School of Electrical Engineering, Politehnica University Bucharest, 060042 Bucharest, Romania

**Keywords:** Biotechnology, Engineering

## Abstract

We demonstrate a proof of concept for magnetically-driven 2D cells organization on superparamagnetic micromagnets fabricated by laser direct writing via two photon polymerization (LDW via TPP) of a photopolymerizable superparamagnetic composite. The composite consisted of a commercially available, biocompatible photopolymer (Ormocore) mixed with 4 mg/mL superparamagnetic nanoparticles (MNPs). The micromagnets were designed in the shape of squares with 70 µm lateral dimension. To minimize the role of topographical cues on the cellular attachment, we fabricated 2D microarrays similar with a chessboard: the superparamagnetic micromagnets alternated with non-magnetic areas of identical shape and lateral size as the micromagnets, made from Ormocore by LDW via TPP. The height difference between the superparamagnetic and non-magnetic areas was of ~ 6 µm. In the absence of a static magnetic field, MNPs-free fibroblasts attached uniformly on the entire 2D microarray, with no preference for the superparamagnetic or non-magnetic areas. Under a static magnetic field of 1.3 T, the fibroblasts attached exclusively on the superparamagnetic micromagnets, resulting a precise 2D cell organization on the chessboard-like microarray. The described method has significant potential for fabricating biocompatible micromagnets with well-defined geometries for building skin grafts adapted for optimum tissue integration, starting from single cell manipulation up to the engineering of whole tissues.

## Introduction

2D organization of cells is of primary importance for identifying rare tumorous cells or stem cells among a wide population, for long time monitoring of individual cells, for studying the communication between cells, for cell therapy and reprogramming^1^. Moreover, this first level of organization can be efficiently used as a starting point for tissue engineering^2–4^. Fibroblasts are spindle-shaped cells providing the primary source of extracellular matrix (ECM) proteins that act as scaffolds for the cells and determine the cells phenotype and functions^5^. They also play key roles in the immune response to tissue damage by initiating inflammation in the presence of attacking microorganisms^6^. Moreover, the fibroblasts are used in cell therapy for the treatment of burns, diabetic wounds, scars, and aging skin^7^. To date, 2D organization of fibroblast cells has been achieved with microfluidic devices fabricated from multilayered SU-8 molds combined with PDMS – based soft UV lithography, which is a multi-step and complicated procedure^6^.

A promising approach for cellular organization is to control the cellular behavior trough forces exerted by magnetic fields^8^. The magnetism is being used to remotely guide, assemble and stimulate various cell types^8–15^ and for engineering tissue sheets^16^, cellular aggregates^17^ and tubular structures^18,19^. Remote cells manipulation using high gradient magnetic fields created across the cells bodies by miniaturized magnetic devices has been recently demonstrated^20^. When placed in an external static magnetic field, the magnetic field in the vicinity of these micromagnets varies at the same scale as the cell size and influences the cellular behavior through high magnetic field gradients^21–23^. To date, arrays of micromagnets producing magnetic field gradients up to 10^11^ T/m have been used to trap arrays of Jurkat cells^1^ and cells functionalized with magnetic nanoparticles^24^. Patterned micromagnets have been also used for guiding the behavior of mesenchymal stem cells^10,23^. A ferromagnetic micromagnet-integrated microfluidic system has been recently developed for enhanced detection of circulating tumor cells labeled with magnetic nanoparticles^25^.

Starting from 1996, the scientists state that the human tissues are at the border between diamagnetic and paramagnetic state (susceptibility ~ − 11.0 × 10^–6^ to − 7 × 10^–6^), very close to water susceptibility (-9.05 × 10^–6^); it is also known that the susceptibility of water is mostly due to Langevin diamagnetism, but there is a small contribution (~ 10%) from van Vleck paramagnetism^26^. Very limited magnetic susceptibility information is available for the biological samples at their physiological conditions, which means in solution and at body temperature^27^. For example, deoxyhemoglobin is paramagnetic, but oxyhemoglobin is diamagnetic and therefore,various states of red blood cells have different magnetism; furthermore, the mass susceptibility of human nasopharyngeal carcinoma CNE-2Z cells measured recently showed a strong paramagnetic component at low temperature, indicative of paramagnetic components in the cells^27^. Moreover, the mass susceptibility of cytoplasm was found to be (9.888 ± 0.6) × 10^–9^ m^3^ /kg, which means that the cytoplasm of CNE2Z cells is paramagnetic as opposed to diamagnetic nucleus^27^. In conclusion, the biological cells and tissue are diamagnetic with susceptibility very close to that of water, but they also contain a paramagnetic component, most probably in the cytoplasm. To our knowledge, no information about magnetic susceptibility of fibroblast cells is available.

Moreover, the use of micromagnets for cell patterning faces several drawbacks. One is that the fabrication methods generally require multi-step and complicated procedures. For example, arrayed nickel micromagnets for screening and molecular analysis of single circulating tumor cells were fabricated by patterning of a photoresist by photolithography, followed by thermal deposition of a thin chromium layer as an adhesion layer, of a thin-film of nickel layer and finally by photoresist removal^25^. Nd-based micromagnets were fabricated by patterning Si pillars using lithography and deep reactive ion etching, followed by high rate triode sputtering of Ta/NdFeB/Ta trilayers that were uniformly coated with a parylene layer for biocompatibility^10^. Another issue is that, for achieving magnetic trapping of cells, the dimensions of the magnets should range from millimeter down to micrometric range^28^. In this regard, a major bottleneck is scaling down the magnetic systems for increasing the magnetic field gradients. An additional drawback is that, for being moved using external magnets, the cells first have to be magnetized by the internalization of MNPs, which raises problems related to nanoparticles toxicity and removal^29^.

In this context, we propose a simpler and versatile method for producing superparamagnetic microarrays by using laser direct writing via two photons polymerization (LDW via TPP). The novelty of our work relies in the demonstration of a proof of concept concerning the magnetically driven of cells on 2D heterostructures composed by alternative superparamagnetic and non-magnetic micro-squares fabricated by LDW via TPP of Ormocore/magnetic nanoparticles composite.

The goal of the present study was to manipulate the 2D organization of fibroblasts trough the magnetization of superparamagnetic micromagnets exposed to a remotely controlled static magnetic field. To this end, LDW via TPP was used for the polymerization of nanocomposites containing a biocompatible photopolymer (Ormocore) mixed with superparamagnetic nanoparticles (MNPs), the will be further denominated *Ormo/MNPs*. The micromagnets were designed in the shape of squares with lateral dimension of 70 µm. To minimize the role of topographical cues in cell organization, we built 2D microarrays similar with a chessboard, where the superparamagnetic micromagnets alternated with non-magnetic areas of identical lateral size as the micromagnets and made by Ormocore (further named *Ormo*). The 2D microarrays were investigated in terms of morphology and chemical composition. The presence of MNPs at desired locations in the micromagnets and the homogeneity of their spatial distribution were demonstrated. The potential of the superparamagnetic micromagnets to control the 2D organization of nanoparticle-free fibroblasts by exposure to a static magnetic field was assessed.

The proof of concept demonstrated by this study regarding the magnetically-driven 2D organization of fibroblasts on superparamagnetic micromagnets exposed to an external static magnetic field surpasses the limitations of the above described existing approaches. First, for fabricating the superparamagnetic micromagnets, we used LDW via TPP, which is a simple, versatile and robust method able to produce complex geometries with high spatial accuracy and reproducibility^30,31^. Second, we operated with MNPs-free fibroblasts cells, eliminating the issue of MNPs toxicity and removal.

## Results and discussions

One of the important aspects concerning the use of micromagnets to guide the cells behavior is their size and positioning^25^: the thickness has to be as low as possible to reduce the physical damages of the cells, the lateral dimensions determines the lateral magnetic effective range of the micromagnets, while the spatial periodicity is important for the spatial distribution of the seeded cells. Here, by taking advantage of the versatility of the structures that can be fabricated by LDW via TPP, we produced 2D microarrays of squared superparamagnetic microstructures less than 10 µm in height compared with the surroundings and having the lateral dimension of 70 µm, which is very close of a standard dimension of a fibroblast cell.

A reason for choosing LDW via TPP over other fabrication techniques relies on the already demonstrated fact that the magnetic interaction benefits greatly from the reduction of the magnets dimensions. So far, nanolithography and thermomagnetic lateral patterning have been used to fabricate micro and nano magnets^10^, but these approaches are not suitable for polymers processing while LDW via TPP is designed for polymers processing with submicrometric resolution. It is known that the cells are better manipulated if they are exposed to high field gradients, where the magnetic field produced by micro-magnets change significantly in value and direction across the cell body^10^. However, the techniques used for micromagnets fabrication involved multistep procedures, often not suitable for processing polymers/magnetic nanoparticles composites owing to their invasive character that lead to polymer degradation or even nanoparticles overheating and therefore degradation of their magnetic properties. Moreover, until now the fabrication of high quality, biocompatible micromagnets has been a bottleneck for scaling down magnets towards further increase the field gradients. This issue is also addressed by using LDW via TPP that allows precise and reproducible patterning at submicrometric scales^1^. Concerning the use of a more “popular” 2D patterning method that is UV lithography^32^ instead of LDW via TPP, we detail a comparative analysis of the shortcomings and advantages of both techniques in the *Methods* section.

**Figure 1:**
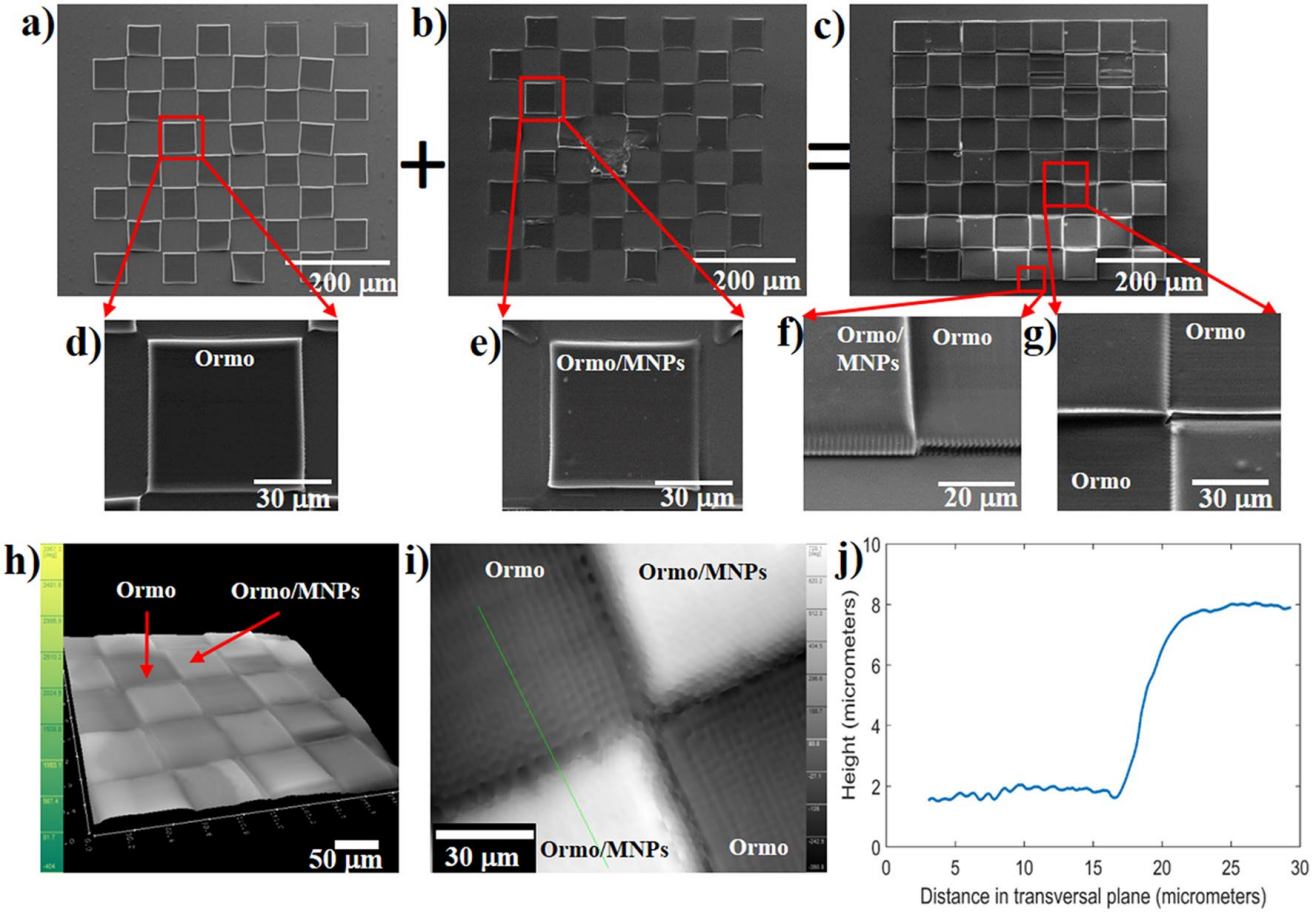
Scanning electron micrographs of 2D microarray fabricated by LDW via TPP: (**a**) *Ormo* squares; (**b**) *Ormo/MNPs* squares; (**c**) *Ormo* superposed with *Ormo/MNPs* squares forming the chessboard-like 2D microarray; (**d**) inset from (**a**); (**e**) inset from (**b**); (**f**) inset from (**c**) showing *Ormo/MNPs* square (left) and *Ormo* square (right); (**g**) Inset from (**c**) showing the intersection of 4 squares from the 2D microarray; DHM images of 2D microarray: (**h**) 3D reconstructed hologram; (**i**) phase shift 2D map at the intersection of 4 squares; (**j**) line profile of the height difference between adjacent *Ormo/MNPs* and *Ormo* squares along the green line from (**i**).

The 2D microarrays comprised *Ormo/MNPs* and *Ormo* squares alternating like on a chessboard (Fig. 1a,b,c). They were obtained by superposing *Ormo* (Fig. 1a,d) and *Ormo/MNps* squares (Fig. 1b,e), as described in Fig. 1a,b,c and fabricated as described in the *Experimental* section.

Although each fabrication step used the same writing parameters (specified in the *Experimental* section), the results indicated different heights for for the *Ormo/MNPs* and *Ormo* squares (Fig. 1f,g). The first reason for this height difference is that the *Ormo/MNPs* composite has slightly different monomer density and refractive index than *Ormo*, which resulted in the formation of a larger volume pixel. The second reason is that for the first fabrication step (*Ormo/MNPs* squares), the autofocus software was able to indicate an approximate position of the substrate surface relative to the focus of the incident laser beam, whereas for the second fabrication step (*Ormo* squares), the autofocus step did not show any indication with respect to surface position. Instead, it was aligned manually. The height difference between adjacent *Ormo/MNPs* and *Ormo* squares was computed using the reconstructed images from DHM (Fig. 1h, i). An example of a profile line is displayed in Fig. 1j. The height difference between two adjacent *Ormo* and *Ormo/MNPs* squares was estimated to be around 6 µm (Fig. 1j). The height difference measured based on the SEM image from Fig. 1f returned a similar result.

The irregular heights and gaps between the magnetic and non-magnetic squares observed in the SEM images are related to the XYZ positioning of the samples in the Nanoscribe system used for LDW via TPP. The XY repositioning accuracy was ± 2 µm. Because we had to remove the samples between the first and the second steps (for drop casting the second material i.e. *Ormo* on the first set of squares i.e. *Ormo/MNPs)*, the samples positioning between the first and second steps was achieved manually, using the laser spot size as a reference. This caused some small gaps between the *Ormo* and *Ormo/MNPs* squares. The differences in height between the *Ormo* and *Ormo/MNPs* squares are caused by the sample positioning on the Z axis (thus on focusing accuracy) that was of about 2 µm. The focusing procedures for each step are detailed in the *Methods* section.

Apart from the low fabrication time and costs, LDW via TPP offers another advantage, which is 3D printing. It is true that, in the present study, we demonstrated the proof of concept concerning the magnetically-driven manipulation of cells choosing simple structures in the shape of 2D microarrays. However, for tissue engineering there is need to control the cells to grow in 3D geometries similar with natural tissues or organs. Therefore, the next step would be to reach magnetically-driven cells manipulation in 3D structures. For this, we will have to build 3D constructs with alternating superparamagnetic and non-magnetic components that will guile the cells to grow in 3D when exposed to static magnetic fields. Other available techniques, such as electron beam lithography or UV lithography are not suitable for such a purpose. This first is too invasive for processing the sensitive materials like polymers while also preserving the superparamagnetic properties of the nanoparticles, the second does not allow facile 3D printing of such materials. For 3D structuring of polymeric and even composite materials, LDW via TPP technique has unique advantages, with practical no constraints regarding the desired geometry along with full reproducibility and high spatial accuracy for the imprinted structures. Moreover, the height of our parallelepiped shaped structures will affect the strength of the binding force between magnetic structure and cell therefore the third dimension of our structures is also important.

**Figure 2:**
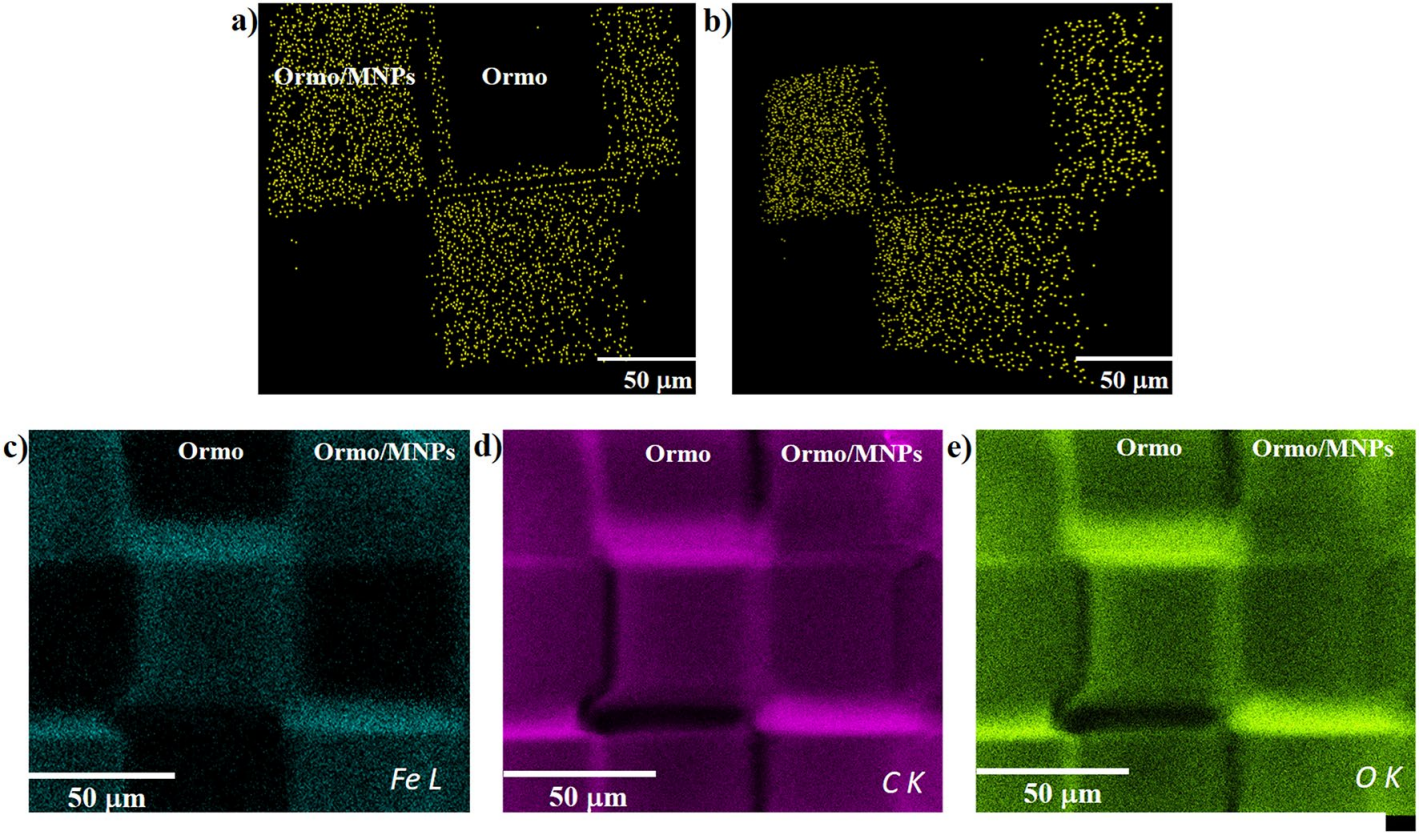
Images of 2D microarray obtained by enhanced dark field microscopy using the Cytoviva 3D module (MNPs represented as yellow dots (false colors)): (**a**) top view; (**b**) tilted view; (**c**–**e**) EDX mapping of iron, carbon and oxygen.

The location and the spatial distribution of the MNPs were monitored by enhanced dark field microscopy (Fig. 2a,b). The MNPs were localized by running the "just locate nanoparticles" routine. The coordinates of the MNPs centers were located inside 80 slices acquired at a distance of 100 nm between them. The MNPs appear as yellow dots (false colors).

The MNPs appear embedded in the superparamagnetic *Ormo/MNPs* micromagnets and have a homogeneous distribution except the edges of the squares, where it appears that the enhanced dark field microscopy and the EDS mapping techniques returned somehow contradictory results. To explain this difference, we will go into more details about the way that the two detection techniques work.

The CytoViva Ultra Resolution Imaging used for locating the MNPs in our study is a standard dark field microscope working in transmission. The dark field microscopic method is a standard method based on images formed by collecting the scattered light from nanometric details of the sample^33^. The CytoViva enhanced dark field microscopy system is used since 2007 to visualize nonfluorescent silver and aluminum nanoparticles inside cells^34^. Several papers are very recently published where CytoViva system was employed to study the phototoxicity and localization of nonfluorescent nanoparticles in wheat plants^35^ and to localize nanoparticles inside electrospun nanofibers^36^.

The CytoViva system is based on an Olympus microscope with specific improvements that increase the imaging performance: the illumination system, the condenser and the camera detectors. A high-aperture cardioid annular condenser is illuminated through a liquid light guide that focuses the light onto an annular entrance slit. The annular A-condenser produces a narrow diffraction pattern of the sample details, resulting in spatial resolution well below 90 nm^37^. This high spatial resolution allowed us to detect single MNPs and MNPs agglomerations that scattered the incident light. These appear as bright pixels in the recorded images. These pixels do not constitute the shapes of the MNPs, instead they indicate their presence and distribution in the sample. The routine delivered by the CytoViva system producers enabled us to replace the bright pixels with yellow pixels (false colors), as displayed in Fig. 2a,b. The procedure for “just locate nanoparticles” was carried out accordingly to the manufacturer’s specifications and uses intensity differences in the whole stack to separate nanoparticles from other structures based on scattering intensities; the routine counts these objects and gives a separate 3D stack with the center of the nanoparticles^33^.

The MNPs distribution at the edges of the *Ormo/MNPs* squares appears inhomogeneous most likely because of some difficulties we encountered in the deconvolution operation. The CytoViva enhanced dark field microscopic system uses several routines that influence the sharpness of the samples edges^33^. One routine is Blur Sigma that applies a blurring routine to deconvolve data to smooth edges. Another routine is Z-sharpen after deconvolution that applies a Z-stack sharpening routine to all stacks. An important parameter for image deconvolution is the maximum number of iterations. As expected, more iterations allow more details to be deconvolved. In our experimental conditions, the structural complexity of the samples and computational constraints forced us to run a limited number of iterations. This influenced the Z-stack sharpening routine and most probably caused a lack of data i.e. fewer MNPs in the Z-stacks acquired at the edges of the *Ormo/MNPs* squares. All these have the started point in the experimental image acquisition, where additional phenomena such as scattering and diffraction on these high and narrow edges occur in the small gaps between the squares.

The chemical composition of the 2D microarrays was determined by EDS mapping. Iron was homogeneously distributed exclusively on the superparamagnetic *Ormo/MNPs* micromagnets, whereas in the non-magnetic *Ormo* squares no traces of iron were detected (Fig. 2c). This results is in good agreement with the data returned by dark field microscopy from Fig. 2a,b. Moreover, the EDS results confirmed that the nanoparticles from the superparamagnetic *Ormo/MNPs* micromagnets are iron-based nanoparticles and thus possess magnetic properties. The presence of carbon and oxygen was detected all over the 2D microarray (Fig. 2d,e).

The EDS mapping images are the proof that Fe atoms exists over all magnetic composite surface i.e. *Ormo/MNPs* squares, while no signal was collected from the non-magnetic *Ormo* squares. To be more specific about the EDS results, in Table 1 we show the elemental composition of an *Ormo/MNPs* square as obtained based on EDS spectra. The level of Fe doping in the composite (of about 4 mg/mL, as described in the *Materials and Methods* section) is very close to the quantitative limit for trace elements, but still matches with the elemental composition (4.91 wt% for Fe) detected experimentally. The estimated depth from which we collect characteristic X-ray photons at 5 kV acceleration voltage is about 0.3 μm.

**Table 1:** Elemental composition of *Ormo/MPNs* square determined by EDS.

Element	Weight (%)	Atomic (%)	Error (%)
C K	62.57	71.07	3.47
O K	32.52	27.73	4.66
Fe L	4.91	1.20	3.49

A controversial aspect is related to the origin of the stronger Fe signals arising from the edges of the *Ormo/MNPs* squares. To explain this effect, we mention that EDS spectra are influenced by surface roughness that affects the local absorption path changing the X-ray yield and leading to errors as high as 10–20%^38^. The difference in square heights between *Ormo* and *Ormo/MNPs* is of several μm, much more than 50 nm recommended to reduce the geometric effects to a negligible level^39^. Furthermore, in EDS the intensity of X-ray signal is stronger for the surfaces that are facing the detector and weaker from surfaces that are orientated to other directions, for all characteristic energies^40^. This indicates that the different signal intensities observed in our samples is in close relationship with their geometry. Specifically, the difference in X-ray signals corresponding to Fe from the center and some of the edges of the *Ormo/MNPs* squares comes from the different spatial orientation of these areas (Fig. 1), in the way that the upper edge in Fig. 2c is facing the detector and the X-rays will go directly to the detector without any absorption in material, while at the lower edge the X-ray signal is lowered by absorption through composite. This topological effect is significantly reduced on the left and right edges, as those edges are facing the detector in a similar manner. The trend is similar for all 3 elements (C K, O K, Fe L) probed by EDS mapping.

**Figure 3:**
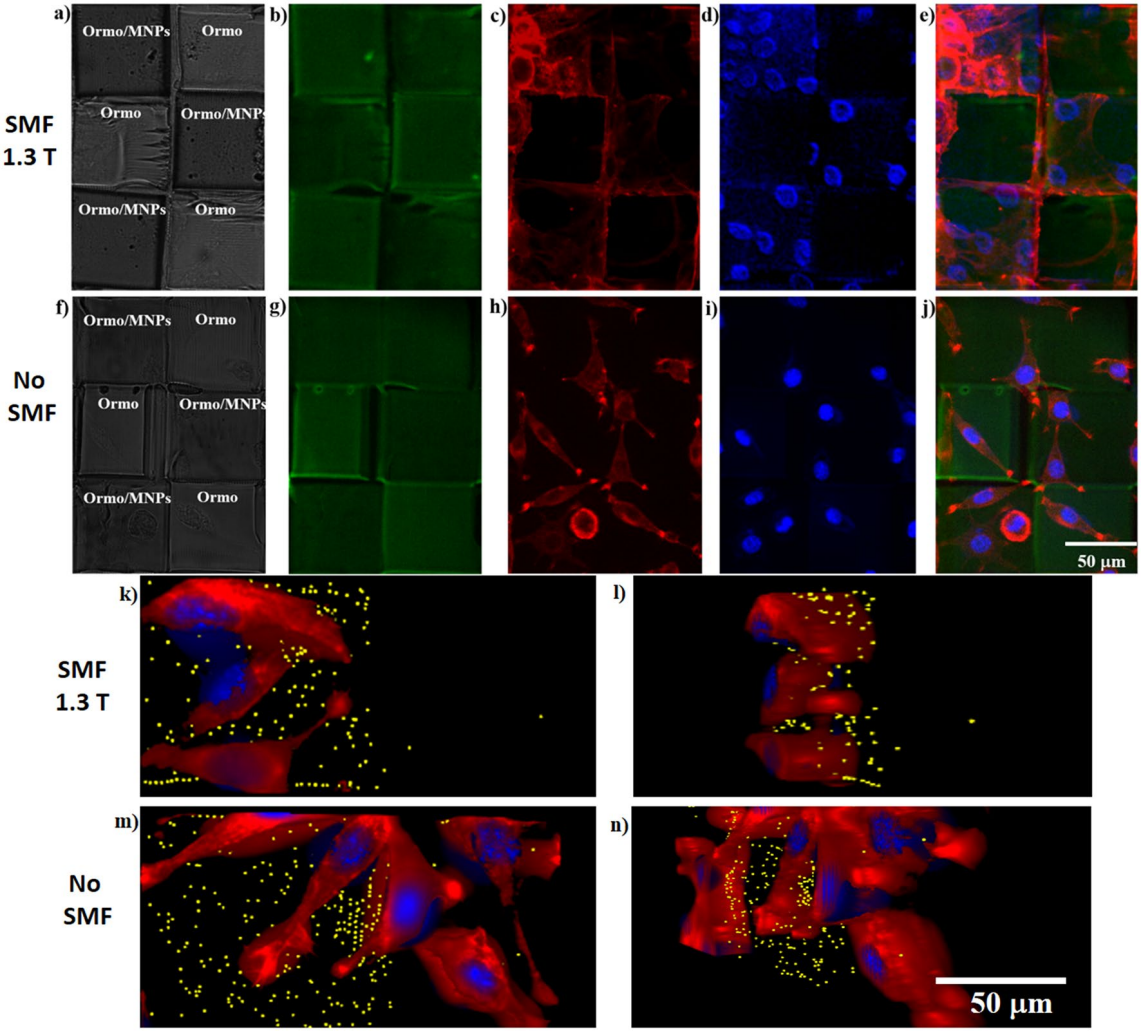
2D microarrays: (**a**, **f**) Optical images; (**b**, **g**) Green autofluorescence; Representative fluorescence microscope images of fibroblasts seeded on 2D microarrays: (**c**, **h**) cytoskeleton (red) visualized by staining with F-actin; (**d**, **i**) nuclei (blue) visualized by staining with Hoechst; (**e**, **j**) overlapped fluorescence images of 2D microarrays, cells cytoskeleton and nuclei; (**k**, **m**) 3D top and (**l**, **n**) 3D tilted views of cells nuclei (blue), cytoskeleton (red) and centers of MNPs and agglomerations of MNPs (yellow), imaged by enhanced dark field microscopy. The samples were imaged in the presence (**a**–**e**, **k**, **l**) and absence (**f**–**j**, **m**, **n**) of SMF. All cells were imaged after 1 day of cell culture.

Finally, we tested the efficiency of the superparamagnetic micromagnets for 2D organization of fibroblasts. We first imaged the 2D microarrays by optical (Fig. 3a,f) and fluorescence microscopy (Fig. 3b,g) respectively. The optical images allowed us to identify the superparamagnetic *Ormo/MNPs* micromagnets (darker due to the presence of the MNPs) from the non-magnetic *Ormo* squares (brighter).

After the fibroblasts were seeded on the 2D microarrays, their behavior was investigated after 1 day of continuous SMF stimulation with 1.3 T. The fluorescence from the cells cytoskeleton (Fig. 3c,h) and nuclei (Fig. 3d,i) was imaged. For the samples exposed to SMF, the superparamagnetic *Ormo/MNPs* micromagnets exerted a clear influence on the cellular attachment comparative to similar samples unexposed to a magnetic field. Following SMF exposure, the cells attached exclusively on the superparamagnetic *Ormo/MNPs* micromagnets, while the non-magnetic *Ormo* squares were free of cells; on the other hand, the cells from the samples that were not exposed to SMF exhibited a random and uniformly distributed attachment on the whole surface, with no preference for superparamagnetic or non-magnetic areas (Fig. 3c,d versus Fig. 3h,i). A second interesting finding concerns the cells morphology for SMF exposed versus unstimulated samples. The cytoskeletons of the cells exposed to SMF expanded until they covered the entire surface of the superparamagnetic *Ormo/MNPs* micromagnets, with poorly visible focal adhesion points (Fig. 3c,e). On the contrary, the unstimulated cells showed the traditional spindle-like cell shape, with pronounced focal adhesion points situated mostly on the edges of both superparamagnetic and non-magnetic areas (Fig. 3h,j). The 3D images of cell-seeded on the chessboard-like 2D microarray obtained by enhanced dark field microscopy confirm the above results (Fig. 3k-n).

Beside the influence of the SMF on cellular morphology and attachment, there are additional factors that influence the cells adhesion on a substrate, including the chemical composition, surface roughness, topography and wettability^41^. In our experimental conditions, surface chemistry of the superparamagnetic *Ormo/MNPs* and non-magnetic *Ormo* squares is similar with the exception of the presence of MNPs that were physically mixed with the photopolymer, without chemical interactions that could give rise to additional compounds. What differed substantially between the superparamagnetic *Ormo/MNPs* and non-magnetic *Ormo* areas was surface topography. Namely, the surface of the *Ormo/MNPs* areas showed some small bumps of submicronic size that most likely represent the MNPs embedded in the photopolymer at close proximity of the sample surface (Fig. 1e,f,g). However, the fact than in the absence of the SMF, the cells were uniformly distributed on the entire 2D microarray shows that this small difference in surface structuring between the superparamagnetic and non-magnetic areas is not a major factor for the cellular attachment.

As for possible explanations for our experimental findings, we advance the idea that when placed in an external magnetic field, the superparamagnetic *Ormo/MNPs* micromagnets were magnetized and generated a localized strong magnetic field that enhanced the attractive interactions between cells and the substrate^25,30^. As we already demonstrated for recently developed 3D superparamagnetic structures^30^, the *Ormo/MNPs* polymerized composite with 4 mg/mL MNPs concentration gives rise to field gradient between two adjacent MNPs of the order of 3 × 10^4^ T/m. In this study, we employed an identical MNPs concentration in Ormocore, i.e. 4 mg/mL as in^30^. Even though the structures had different architectures and scope, this explains the fact that we succeeded to create high enough magnetic field gradients which enabled magnetically-driven 2D organization of fibroblasts on the chessboard-like microarrays.

These experimental findings are consistent with the findings of Zablotskii et al. (2010) that manipulated stem cells on NdFeB squared micromagnets produced by electron beam lithography and deep Reactive Ion Etching^10^. The study showed that high static magnetic field gradients generated by micromagnet arrays were able to assist the cells migration to the areas with the strongest magnetic field; specifically, in SMFs with gradients above 10^4^ T/m the magnetic force magnitudes are comparable with the gravitational forces and affect the cell machinery^10^. The authors report that, after several days of incubation, the cells grew on the tops of the micromagnets and their spatial arrangement reflected the geometry of the underlying micromagnet arrays. The authors ascertain their findings to the fact that, in the presence of high magnetic field gradients, the cells have a paramagnetic behavior, most likely because they are less diamagnetic than the surrounding medium; in this case, the magnetic forces attract the cells towards the areas with the highest magnetic field gradients and finally the cells expand on the whole surface of the micromagnets. According to^30^, in our experimental conditions the field gradient between two adjacent MNPs is of the order of 3 × 10^4^ T/m, high enough to induce similar effects on the chessboard-like microarray.

Despite the intensive research on the use of magnetic materials in bio-medical applications, until present the precise influence of a magnetic field at cellular level is not completely revealed. A particularly interesting case is when a living cell interacts with a magnetic field gradient of similar size to itself. Such an effect was for example reported on NdFeB micromagnets arrays seeded with MNPs-free mesenchymal stem cells that adhered to the magnetic surfaces and elongated in directions parallel to the edges of the micromagnets^10^. Of course, in addition to the cell shape modifications, the phenomena occurring at cellular level under the action of magnetic forces are likely far more complex, affecting cell functions such as membrane deformation, endocytosis, exocytosis, motility and cytokinesis. Equally important, one must remember that, for being viable, the cells in the magnetic structures must connect to each other. Therefore, for creating functional tissue, further investigations about cell migration and cell networking for longer culture times are needed.

A particular point would be to clarify in what degree the novelty of the approach proposed by our study distinguishes from the original findings reported by previous works. It is already known that the multimaterial 3D micro-printing was reported years ago. For example, in^46^ the authors manufactured 3D microporous multicomponent polymer scaffolds out of different organic–inorganic substances. The goal of those composite constructions was to provide biological functionalities such as biostability and possibility to micro/nano-pattern the surface with bioactive materials such as proteins. The concept and the purpose of that study differ considerably from the methodology and scope of our work. On reason is that in the present study we present heterostructures where the magnetic properties are accounted. To this end, we joined a first magnetic part with a second nonmagnetic part to produce a chessboard-like microarray for 2D magnetically driven cells manipulation. Another reason is that our goal differ from that of the study reported in^46^. Our objective was to test de ability to manipulate the cells growth in 2D on superparamagnetic structures by remotely applied static magnetic fields. The outcome of our 2D approach is useful for systematic in vitro studies of cells behavior in static magnetic fields. Equally important, our findings provide a framework for further developments of LDW via TPP of 3D heterostructures with alternating magnetic and nonmagnetic parts for engineering whole tissue or organs by exposure to static magnetic fields.

Related to a previously published study on functional magnetic microstructures^47^, we bring the following arguments in favor of originality of our work. First of all, in^47^ the authors used another technique and other materials for producing the magnetic structures than those employed in our study. Specifically, that study reports on the use of low one-photon absorption direct laser writing for the fabrication of three-dimensional magnetophotonic devices on a photocurable homogeneous nanocomposite consisting of magnetite nanoparticles and a commercial SU8 photoresist^47^. The principle of two photons absorption used in our study relies on different absorption phenomena, namely we used a fs laser 800 nm instead of the 532 nm cw laser used in^47^. Second of all, the properties of the photopolymerizable materials used in^47^ differ significantly for the materials we used: studies on adult myogenic stem cell proliferation showed that Ormocore photopolymer is applicable for biomedical tissue engineering practice^48^, whereas the biocompatibility of SU-8 used in^47^ needs further improvements by surface and chemical treatments^49^. Third, in^47^ the authors report exclusively on three-dimensional submicron mechanical magnetophotonic devices and some of their potential applications, with no proof on the design, fabrication or use of such structures for magnetically driven manipulation of cells.

## Conclusions

In this work we demonstrated for the first time a proof of concept for magnetically-driven 2D cells organization on superparamagnetic micromagnets exposed to an external static magnetic field. We designed and fabricated 2D heterostructures in the form of chessboard-like microarrays, where superparamagnetic Ormocore/MNPs micro-squares alternated with non-magnetic Ormocore micro-squares. For structures fabrication, we relied on the unique advantages of LDW via TPP, specifically high spatial resolution and reproducible imprinting, preservation of the superparamagnetic properties of the magnetic nanoparticles and unique potential for further 3D development of the heterostructures for magnetically-controlled tissue engineering.

The micromagnets were integrated in a 2D microarray formed by superparamagnetic and non-magnetic areas that alternated like on a chessboard. Both superparamagnetic and non-magnetic areas were fabricated by laser direct writing via two photons polymerization (LDW via TPP) of a superparamagnetic composite (Ormocore/MNPs) and of a non-magnetic photopolymer (Ormocore) respectively. Both types of areas were designed in the shape of squares with lateral dimension of 70 µm. When no magnetic field was applied, the cells attached randomly on the entire chessboard-like 2D microarray, showing no preference for superparamagnetic or non-magnetic areas. In the presence of a static magnetic field of 1.3 T, the cells preferentially attached on the superparamagnetic micromagnets, resulting an accurate 2D organization of the fibroblasts on the chessboard-like microarray. This approach has significant potential for building skin grafts with well-defined geometries adapted for optimum tissue integration. In perspective, the capability of LDW via TPP to produce complex superparamagnetic 3D structures with high spatial accuracy and reproducibility has significant potential for “engineering” whole tissues or organs.

## Methods

### Materials

The superparamagnetic micromagnets were fabricated from nanocomposites containing a biocompatible commercially available photopolymer named Ormocore (Microresist Technology GmbH) and superparamagetic nanoparticles (MNPs) in 4 mg/mL concentration. This specific concentration was previously established by us as a tradeoff between the possibility to perform the photopolymerization on the Ormocore/MNPs composite and the ability to obtain high enough magnetic field gradients that induce changes at cellular level^30^. The photopolymer and the developer (OrmoDev) were purchased from Microresist Technology GmbH.

The unpolymerized Ormocore/MNPs composite was homogenized by 1000 W ultrasonicator at 20 kHz (Hielscher Ultrasonics GmbH, Model UIP1000hdT) for 30 s. The superparamagnetic nanoparticles with 4.9 ± 1.5 nm diameters and maghemite structure (gamma-Fe_2_O_3_) were produced by laser pyrolysis as described in^42,43^. The MNPs from the polymerized Ormocore/MNPs composite preserved their superparamagnetic behavior and had a specific magnetization of about 17 emu/g^30^.

### Fabrication method

For LDW via TPP we used the Photonic Professional system from Nanoscribe GmbH. The laser source delivered 120 fs pulses, with a repetition rate of 80 MHz, centered on a wavelength of 780 nm. The incident radiation was focused on the samples using an inverted Zeiss microscope, equipped with a 63× microscope objective. *Ormo* and *Ormo/MNPs* squares each having 70 µm lateral dimension were positioned alternatively like on a chessboard. The fabrication process involved two exposure steps. First, the *Ormo/MNPs* composite was drop-casted on glass substrate, followed by LDW via TPP fabrication of the first set of squares i.e. the superparamagnetic micromagnets. For removing the unpolymerized material, the samples were developed through immersion for 3 min in OrmoDev. In the second step, Ormocore was drop-casted on top of the previously formed *Ormo/MNPs* squares. Non-magnetic *Ormo* squares were obtained by LDW via TPP of Ormocore, The unpolymerized material was removed by rinsing in OrmoDev developer. The starting position was set so that the *Ormo* squares filled the gaps between the *Ormo/MNPs* squares fabricated in the first step. Both fabrication steps were made using the same laser writing parameters (90 µm/s velocity and 38.4 mW average power).

The XY repositioning accuracy was ± 2 µm as set by the producer. On the Z axis, the precision in positioning (implicitly the laser focusing) is generally determined by the autofocus option available in the dedicated software of Nanoscribe system and depend on the sample geometry and on the refractive index of the polymerizable material. The Z positioning for the first step (*Ormo/MNPs* composite) was achieved through means of polymer/glass substrate interface localization software (autofocus routine) that uses the image sharpness to detect the surface. The accuracy in Z positioning (the laser focusing) is slightly lower for materials with poor contrast of the refractive index as compared to the glass substrate (such as the case of *Ormocore*). Consequently, for the second polymerization step i.e. *Ormo,* the autofocus algorithms did not work and we had to find manually the focus point. For manual repositioning, the sample was translated on the Z axis until the laser focus superposed with the polymer/glass substrate interface detected by the dedicated software. The accuracy of the positioning on the Z axis was of about 2 µm. In brief, while we find the XY positioning appropriate, the azimuthal accuracy can be accounted for some samples irregularities. We are considering software correction of the azimuth for future experiments.

The fabrication costs of LDW via TPP are debatable, and we would like to argue that, at this particular point of development, LDW via TPP is the cheaper and faster option, especially through the Nanoscribe system we used. LDW via TPP cost is estimated to ~ 17 eur per hour^44^, whereas standard lithographic methods like UV lithography become cheaper for upscaled and not for downscaled structures.

The fabrication methodology for LDW via TPP is also considerably simpler than for UV lithography. A typical LDW via TPP methodology goes as following: fix a glass substrate in a metallic support, drop cast the photopolymerizable material on the glass substrate, laser exposure to print the desired structure in the photopolymerizable materials, and finally the washing away of the non-polymerized material by immersion in an appropriate solvent. Typical, UV lithography adds more complications to both fabrication steps and processing time, involving a pre- and post-processing multi-steps methodology: surface treatment, spin-coating, single or multistep baking, UV exposure, post-baking, and finally development.

Apart from the fabrication time and costs, LDW via TPP offers another advantage, which is 3D printing. It is true that, in the present study, we demonstrated the proof of concept concerning the magnetically-driven manipulation of cells choosing simple structures in the shape of 2D microarrays. However, for tissue engineering there is need to control the cells to grow in 3D geometries similar with natural tissues or organs. Therefore, the next step would be to reach magnetically-driven cells manipulation in 3D structures. For this, we will have to build 3D constructs with alternating superparamagnetic and non-magnetic components that will guile the cells to grow in 3D when exposed to static magnetic fields. Other available techniques, such as electron beam lithography or UV lithography are not suitable for such a purpose. This first is too invasive for processing the sensitive materials like polymers while also preserving the superparamagnetic properties of the nanoparticles, the second does not allow facile 3D printing of such materials. For 3D structuring of polymeric and even composite materials, LDW via TPP technique has unique advantages, with practical no constraints regarding the desired geometry along with full reproducibility and high spatial accuracy for the imprinted structures. Moreover, the height of our parallelepiped shaped structures will affect the strength of the binding force between magnetic structure and cell. Thus the third dimension of our structures is important.

Moreover, LDW via TPP process is easy to use in the dedicated Nanoscribe system described in the Materials and Methods section. This compact system allowed us to optimize the laser exposure parameters such as laser power and laser scan speed, which are now available to start the fabrication of 3D microstructures. On the opposite, switching to UV lithography would require an entire re-parametrization of the whole fabrication process. Moreover, LDW via TPP allows for more complex 3D geometries and smaller spatial features that UV lithography.

Using UV lithography, it would be easy to fabricate a higher quality version of the first set of squares i.e. *Ormo/MNPs*. But the second set of squares i.e. *Ormo* should have a similar height and be closely packed to the first set. That, in turn, provides several challenges for fabrication, and considerably increases the fabrication difficulty when using UV lithography. We would need to go through the pre-processing steps while having the first set of squares already on the substrate. This means casting, spin coating, baking, all while there is already a set of squares on the glass substrate. Turning the second material into a thin film, with a height similar to the first set of squares, might prove to be challenging due to several factors: height homogeneity, edge effects due to surface tension, formation of air pockets, surface adhesion, different dilatation coefficients during the baking steps. The UV exposure step should require that provided masks are well aligned or the positioning is well defined for maskless versions. Post-baking and development involved in UV lithography may also require additional optimization steps, due to the dilatation coefficients of the two materials. Pre- and post-baking processes might also induce unwanted structural damage to the first set of squares (cracking and exfoliation). All these additional steps and parameters add to the costs of using UV lithography instead of LDW via TPP, mostly through means of the required fabrication and optimization time.

### Characterization

#### Scanning electron microscopy (SEM)

The morphology of the chessboard-like 2D microarray was investigated by Scanning Electron Microscopy (SEM, FEI InspectS model). Prior to examination, the samples were coated with a 10 nm layer of gold.

#### Digital holographic microscopy (DHM)

The samples (without any special preparation, only covered with a water drop) were investigated in a DHM experimental setup based on a Mach–Zehnder interferometer in off axis configuration, working in transmission^45^. A 100× objective was inserted in the object arm. The holograms were acquired on a CCD camera (Pike F421C, Kodak sensor 6.4 μm pixel pitch) and reconstructed using Koala dedicated software. The reconstructed 3D images delivered the phase maps of 2D microarrays with different values on *Ormo* and *Ormo/MNPs* squares due to specific optical path shifts introduced along the propagation axis because of the height difference.

#### Enhanced dark-field microscopy (EDFM)

The location and distribution of the MNPs in the chessboard-like 2D microarray were investigated using the CytoViva system (CytoViva Inc., Auburn, AL, USA). The studies were carried out without any prior preparation of the samples and in a nondestructive manner. The dark-field set illuminator was designed for high oblique illumination over the sample to evidence the presence of the MNPs based on the light scattered by its nanometric details. The high signal-to-noise optical performance based on patented illumination system (optical fiber with liquid core and condenser geometry) and particle location routines provided 3D optical images of the sample. The Z stacks images were collected at 100 nm between slices using 60× oil immersion objective on Q-imaging Exi Blue CCD (6.45 × 6.45 μm pixel pitch) at different exposure times. Data recording was carried out in white light for locating the nanoparticles*.* The system was set to represent MNPs in yellow using 2 pixels in the location where they are found automatically by running software dedicated routines.

We collected three stacks of images from the same region of the sample: the first stack was collected with DAPI filter for imaging the cells nuclei, the second stack was collected using the Texas Red filter for imaging the cells cytoplasm and the third stack was done in white light for detecting the MNPs. In each stack, sample was scanned from bottom to top, in 100 nm steps. The acquired images were processed together using dedicated ImageJ plugins, following the procedure delivered by the producer: (1) synchronization of the images and crop of the region of interest; (2) use the point spread function, for simulating the light propagation; (3) deconvolution, to remove the unfocused details; (4) intensity threshold selection, for running the routine that localizes the nanoparticles and for assigning to each MNP a yellow point and 5) using ImageJ special plugins, the three stacks were assembled into one single image where the cells (with nucleus and cytoplasm) and the MNPs were simultaneously visible.

#### Energy-Dispersive X-ray Spectroscopy (EDS)

Was performed at 5 kV acceleration voltage inside Scanning Electron Microscopy (FEI InspectS model) using a 30 mm^2^ SDD detector (EDAX Inc.). The mapping was done using standardless ZAF analysis. The trace analysis for iron provided errors under 1%, as results from standard deviation of 3 measurements on different locations on the sample.

### Biological assessments

#### Cell seeding

The cell seeding protocol was performed according to standard procedures provided by the suppliers^30,31^. L929 fibroblast cells were purchased from ECACC (UK). The cells were cultured in a 25 cm^2^ flask, incubated in an atmosphere of 5% CO_2_ at 37 °C and cultured in Minimum Essential Medium, Merck containing 10% fetal bovine serum (FBS, Biochrom) and 2 mM L-glutamine (complete medium). 100 IU/mL of penicillin/streptomycin was added to the solution. After confluency, the cells were detached with trypsin and seeded on the 2D microarrays. A cell density of 10 000 cells/sample was used. All chemicals were purchased from Sigma-Aldrich, unnless otherwise specified. Prior cell seeding, the samples were sterilized for 1 h under a UV lamp.

#### Cells staining

The staining protocol was performed according to standard procedures provided by the suppliers^30,31^. The cells were stained with Hoechst 33,342 to visualize the nucleus and with Texas Red-X Phalloidin (ThermoFisher Scientific) for F-actin staining. Cells were fixed in 3.7% paraformaldehyde (Chemical Company) for 20 min, permeabilized 15 min with 0,1% Triton X-100 and incubated over night at 4–8 °C with a solution of Texas Red-X Phalloidin. The next day, the cells were washed with PBS and incubated with Hoechst solution for 10 min. After that, the cells were washed twice with PBS, mounted on a microscope slide and visualized under a fluorescence microscope (BX52–Olympus).

#### Static magnetic field stimulation (SMF) of cell-seeded chessboard-like 2D microarray

Nickel-plated NdFeB rectangular magnets (40 × 40 × 20 mm^3^) with residual magnetism of 1.3 T were placed below and in the close vicinity of the samples. Control experiments were carried out on samples without SMF exposure. The magnetic stimulation was applied from 1 to 3 days, given that these timescales of SMF exposure are known to induce changes at the level of cell shape and size^10^.

#### Cells 3D imagining by enhanced dark field microscopy

The same system used for dark field microscopy measurements on the chessboard-like 2D arrays (CytoViva Inc., Auburn, AL, USA) was used for investigating the seeded cells. In this case, a 100 × oil immersed objective was inserted in the experimental setup. The images were acquired with 100 nm between every slice. The number of slices was higher than in the case of cell-free samples *(*around 120 slices, comparing with 80 slices for the samples free of cells). For every zone, the samples were scanned along the propagation axis.
